# The Saturation Effect of Obesity on Bone Mineral Density for Older People: The NHANES 2017–2020

**DOI:** 10.3389/fendo.2022.883862

**Published:** 2022-05-16

**Authors:** Ya Zhang, Jian Pu

**Affiliations:** ^1^ Graduate College of Youjiang Medical University for Nationalities, Baise, China; ^2^ Department of Hepatobiliary Surgery, Affiliated Hospital of Youjiang Medical University for Nationalities, Baise, China

**Keywords:** bone mineral density, osteoporosis, NHANES, obese, body mass index

## Abstract

**Introduction:**

Previous studies have shown that obesity has a positive effect on bone mineral density (BMD). However, excessive obesity is harmful to health, especially in older adults. In addition, it is unclear what body mass index (BMI) and waist circumference (WC) to maintain for the most beneficial BMD in older adults.

**Methods:**

Multivariate logistic regression models were used to investigate the association between BMI, WC, and femoral neck BMD using the most recent data from the 2017–2020 National Health and Nutrition Examination Survey (NHANES). Fitting smoothing curves and saturation effects analysis were also used to determine the association of nonlinear relationships between BMI, WC, and femoral neck BMD.

**Results:**

The analysis included a total of 2,903 adults. We discovered that BMD and WC were positively linked to femoral neck BMD. The favorable associations of BMI and WC with femoral neck BMD were maintained in all subgroup analyses stratified by sex and race, except among Mexican Americans. Furthermore, smoothing curve fitting revealed that the link between BMI and BMD was not only a linear connection, and that there was a saturation point. The BMI saturation value in the femoral neck BMD was 24.3 (kg/m^2^), according to the saturation effect analysis.

**Conclusions:**

In persons over the age of 50, our research found a positive relationship between obesity and BMD, and we also found a saturation value between BMI and BMD. According to this study, maintaining BMI at a moderate level (about 24.3 kg/m^2^) would result in an optimal balance between BMI and BMD in adults over 50 years of age.

## Introduction

Osteoporosis is a long-term disorder marked by reduced bone mineral density (BMD) that affects a huge number of people (1). According to the International Osteoporosis Foundation, more than 30% of women and more than 20% of men over the age of 50 have osteoporosis or osteopenia, putting them at risk for osteoporotic fractures (2). Simultaneously, the prevalence of osteoporosis continues to climb as the population ages and expands (3). Apart from genetics, age, and gender, other variables that affect bone metabolisms, such as food intake and lifestyle, have lately received a lot of attention (4–6). Meanwhile, scientists are working to discover novel ways to prevent and treat osteoporosis.

Obesity is a huge medical problem that affects people all over the world (7). Body mass index (BMI) is commonly used to assess overall obesity, while waist circumference (WC) is used to assess central obesity (8, 9). According to most of the previous studies, obesity and BMD have a strong favorable relationship in older adults (10–12). However, excessive BMI and WC may be associated with other systemic diseases and comorbidities such as atherosclerosis (13), non-alcoholic fatty liver disease (NAFLD), type 2 diabetes (14), and obstructive sleep apnea (15). Therefore, it is crucial to strike a balance between BMI, WC, and BMD. Existing studies are inconclusive on how much BMI and WC is most beneficial for BMD while minimizing the risk of other obesity-related problems. Therefore, we assessed the connection of BMI and WC with BMD in older adults in this study using a comprehensive fraction of individuals aged above 50 years from the National Health and Nutrition Examination Survey (NHANES). We hypothesized that BMI and WC had a saturation point, respectively, and that maintaining BMI and WC at this point would result in the best balance between obesity and BMD.

## Materials and Methods

### Data Source and Study Population

**Figure 1:**
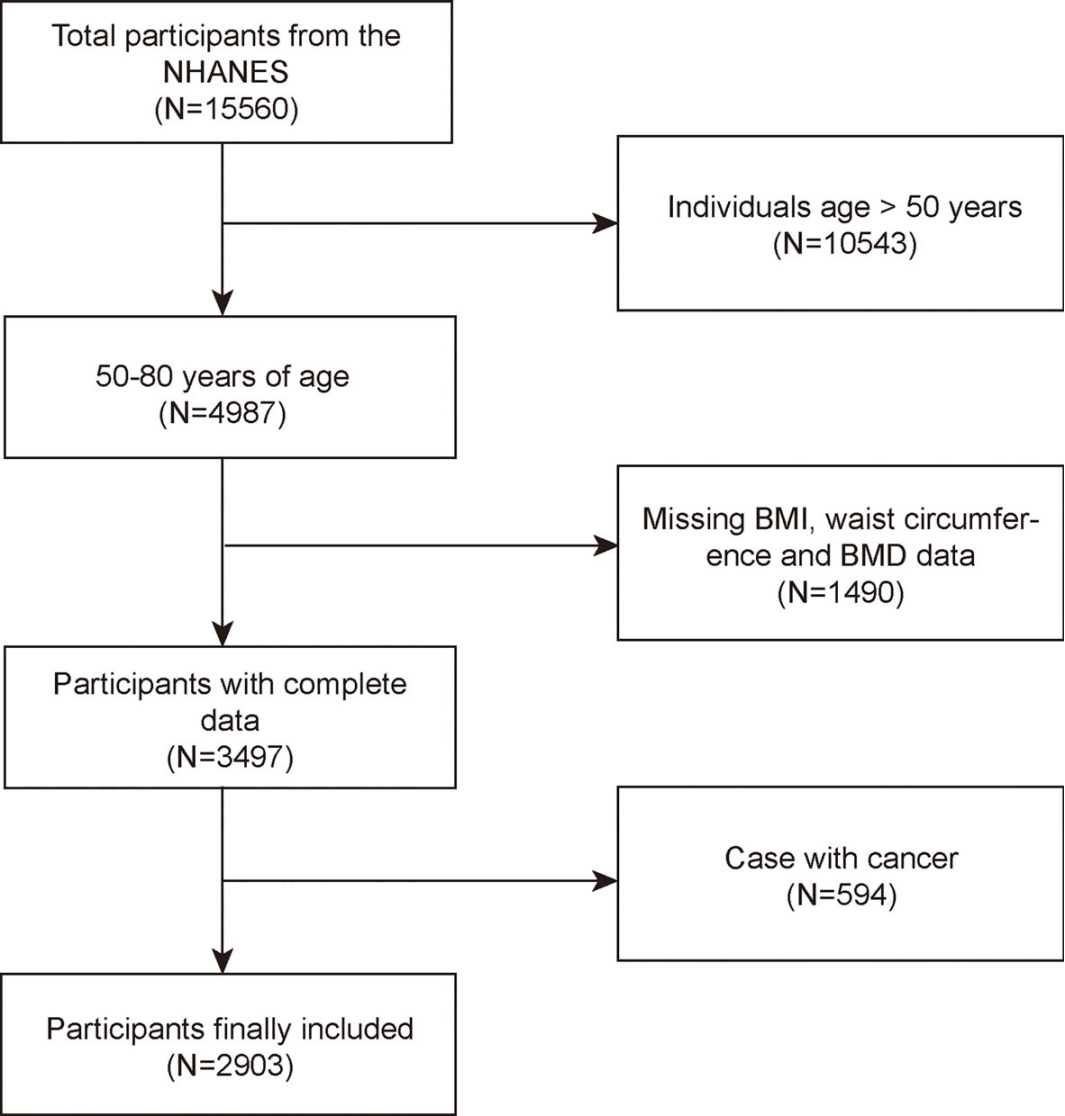
Flowchart of participant selection. NHANES, National Health and Nutrition Examination Survey; BMD, bone mineral density; BMI, body mass index; WC, waist circumference.

The NHANES is a major, continuing cross-sectional survey in the United States that aims to give objective statistics on health issues and address emerging public health concerns among the general public. The NHANES datasets were utilized for this investigation from 2017 to 2020. The participants in the research had to be between the ages of 50 and 80. Among the 15,560 individuals, we excluded 10,543 individuals younger than 50 years, 3,545 individuals with missing BMD, 19 with missing BMI data, 29 with missing WC data, and 594 individuals with cancer diagnoses. Finally, 2,903 people were enrolled in the study (**Figure 1**).

### Ethics Statement

The National Center for Health Statistics Research Ethics Review Board authorized the protocols for the NHANES and obtained signed informed consent. After anonymization, the NHANES data are available to the public. This enables academics to transform data into a study-able format. We agree to follow the study’s data usage guidelines to guarantee that data are only utilized for statistical analysis and that all experiments are carried out in compliance with applicable standards and regulations.

### Study Variables

In the mobile examination facility, certified health workers collected anthropometric data (MEC). Individuals were measured while standing with their arms crossed across their chest. The iliac crests were palpated bilaterally and a horizontal line was drawn just above the uppermost lateral border of the right ilium. After that, the right midaxillary line was drawn. At the point where the two lines crossed, the measuring tape was positioned in the horizontal plane. At the end of the individual’s normal expiration, his or her WC was measured. The BMI was calculated by multiplying the weight by the squared height. Dual-energy x-ray absorptiometry was performed using a Hologic QDR 4500A device and Apex software version 3.2 by qualified radiology technologists to assess femoral neck BMD. Covariates in multivariate models may cause the correlations between BMI, WC, and femoral neck BMD to be muddled. Age, gender, race, education level, activities status, diabetes status, NAFLD status (controlled attenuation parameter values≥274 dB/m were considered suggestive of NAFLD) (16), smoking status, ALT, ALP, AST, total calcium, total cholesterol, direct HDL cholesterol, LDL cholesterol, triglyceride, and serum phosphorus were all covariates in this study. The NHANES website (https://www.cdc.gov/nchs/nhanes/) has a thorough explanation of how these variables are calculated.

### Statistical Analysis

We used R software (version: 4.0.3, Vienna, Austria: R Foundation for Statistical Computing, 2016) and EmpowerStats (version: 2.0, X&Y Solutions, Inc., Boston, MA; http://www.empowerstats.com) for all statistical analyses, with statistical significance set at *p* < 0.05. *p*-values for continuous variables in the population baseline table were calculated by weighted linear regression models, and *p*-values for categorical variables were calculated by weighted chi-square tests. The relationship between exposure factors and BMD was calculated using a weighted multiple regression model. The trend of the effect values is illustrated by calculating *p* for trend from the linear trend test in the regression model. Model 1 did not adjust for variables; Model 2 adjusted for age, sex, and race; and Model 3 adjusted for all covariates listed in **Table 1**. In addition, we performed subgroup analyses, stratifying the variables according to gender, race, and categorization based on the distribution of exposure factors.

**Table 1:** Characteristics of the participants.

Characteristics	Male (*n* = 1,537)	Female (*n* = 1,366)	*p*-value
Age (years)	63.462 ± 8.662	63.446 ± 8.703	0.960
Race (%)			0.200
Non-Hispanic White	33.637	34.919	
Non-Hispanic Black	28.562	25.915	
Mexican American	10.475	9.370	
Other race	27.326	29.795	
*Education level (%)*			0.006
Less than high school	22.837	17.570	
High school	25.569	26.061	
More than high school	51.594	56.369	
*Moderate activities (%)*			0.456
Yes	39.493	38.141	
No	60.507	61.859	
*Diabetes status (%)*			<0.001
Yes	25.374	18.448	
No	70.397	78.331	
*NAFLD status (%)*			<0.001
Yes	51.997	42.113	
No	48.003	57.887	
*Smoked at least 100 cigarettes in life (%)*			<0.001
Yes	57.189	33.602	
No	42.811	66.398	
Borderline	4.229	3.221	
ALT (U/L)	24.143 ± 23.565	19.703 ± 13.894	<0.001
AST (U/L)	23.343 ± 17.798	21.444 ± 10.898	<0.001
ALP (U/L)	78.728 ± 24.659	85.055 ± 27.148	<0.001
Total calcium (mmol/L)	2.318 ± 0.095	2.337 ± 0.099	<0.001
Total cholesterol (mmol/L)	4.702 ± 1.079	5.202 ± 1.062	<0.001
Direct HDL cholesterol (mmol/L)	1.281 ± 0.354	1.568 ± 0.438	<0.001
LDL cholesterol (mmol/L)	2.756 ± 0.928	3.032 ± 1.000	<0.001
Triglyceride (mg/dl)	1.370 ± 1.587	1.213 ± 0.654	0.019
Serum phosphorus (mmol/L)	1.106 ± 0.175	1.187 ± 0.156	<0.001
Waist circumference (cm)	103.307 ± 14.256	99.069 ± 14.739	<0.001
Body mass index (kg/m²)	28.773 ± 5.508	29.530 ± 6.722	<0.001
Femoral neck bone mineral density (g/cm^2^)	0.820 ± 0.145	0.725 ± 0.139	<0.001

Mean ± SD for continuous variables: p-value was calculated by weighted linear regression model.

% for categorical variables: p-value was calculated by weighted chi-square test.

NAFLD, non-alcoholic fatty liver disease.

## Results

### Baseline Characteristics

The demographic and laboratory data of 1,537 men and 1,366 women are presented in **Table 1**. The evaluated BMI, WC, and femoral neck BMD for men were 28.773 ± 5.508 kg/m², 103.307 ± 14.256 cm, and 0.820 ± 0.145 g/cm², respectively. In women, the respective values were 29.530 ± 6.722 kg/m², 99.069 ± 14.739 cm, and 0.725 ± 0.139 g/cm², respectively. The percentage of people with abdominal obesity in each group according to gender and race is shown in **Supplementary Table S1**. Compared to female participants, male participants are more likely to suffer from diabetes and NAFLD. Male participants had significantly higher WC and smoking status, and significantly higher levels of ALT, AST, triglyceride, and femoral neck BMD, while education level, BMI, HDL-cholesterol, LDL, total calcium, total cholesterol, and serum phosphorus were lower than those in female individuals.

### Relationship Between BMI and Femoral Neck BMD

The findings of the multivariate regression analysis are shown in **Table 2**. BMI was strongly positively linked with femoral neck BMD in the unadjusted model [0.009 (0.008, 0.010)]. In addition, this correlation remained significant after adjusting for the covariates in Model 2 [0.008 (0.007, 0.009)] and Model 3 [0.008 (0.007, 0.010)].

**Table 2:** Association between body mass index (kg/m²) and femoral neck bone Mineral density (g/cm^2^).

	Model 1*β* (95% CI) *p-*value	Model 2*β* (95% CI) *p-*value	Model 3*β* (95% CI) *p-*value
Body mass index (kg/m²)	0.009 (0.008, 0.010) <0.00001	0.008 (0.007, 0.009) <0.00001	0.008 (0.007, 0.010) <0.00001
<18.5 kg/m^2^	0.038 (−0.002, 0.077) 0.07327	0.024 (−0.008, 0.056) 0.14941	0.021 (−0.006, 0.051) 0.26542
18.5–25 kg/m^2^	Reference	Reference	Reference
25–29.9 kg/m^2^	0.010 (0.004, 0.016) 0.00062	0.009 (0.004, 0.014) 0.00035	0.004 (−0.004, 0.012) 0.30468
≥30 kg/m^2^	0.006 (0.005, 0.008) <0.00001	0.006 (0.005, 0.008) <0.00001	0.007 (0.004, 0.009) <0.00001
*p* for trend	<0.001	<0.001	<0.001
Subgroup analysis stratified by gender			
Male	0.009 (0.007, 0.010)<0.00001	0.008 (0.007, 0.009) <0.00001	0.008 (0.006, 0.010) <0.00001
Female	0.010 (0.009, 0.011)<0.00001	0.008 (0.007, 0.009) <0.00001	0.008 (0.006, 0.010) <0.00001
Subgroup analysis stratified by race			
Non-Hispanic White	0.008 (0.006, 0.009) <0.00001	0.007 (0.006, 0.009) <0.00001	0.008 (0.006, 0.011) <0.00001
Non- Hispanic Black	0.008 (0.007, 0.010) <0.00001	0.009 (0.008, 0.011) <0.00001	0.009 (0.007, 0.011) <0.00001
Mexican American	0.007 (0.004, 0.009) <0.00001	0.006 (0.003, 0.009) <0.00001	0.002 (−0.002, 0.007) 0.30963
Other race	0.009 (0.007, 0.010) <0.00001	0.008 (0.007, 0.009) <0.00001	0.008 (0.007, 0.010) <0.00001

Model 1: No covariates were adjusted.

Model 2: Age, gender, and race were adjusted.

Model 3: Age, gender, race, education level, activities status, diabetes status, NAFLD status, smoking status, ALT, ALP, AST, total calcium, total cholesterol, direct HDL cholesterol, LDL cholesterol, triglyceride, and serum phosphorus were adjusted.

In the subgroup analysis stratified by gender or race, the model is not adjusted for the stratification variable itself.

On a subgroup analysis stratified by gender, BMI was positively associated with femoral neck BMD in men [0.008 (0.006, 0.010)] and women [0.008 (0.006, 0.010)] in the fully adjusted model with same effect values and confidence intervals. On subgroup analysis stratified by race, the positive relationship between BMI and femoral neck BMD was retained in whites [0.008 (0.006, 0.011)], blacks [0.009 (0.007, 0.011)], and other race [0.008 (0.007, 0.010)] in the fully adjusted model. However, this association became insignificant (*p* = 0.30963) after adjusting all other covariates in Mexican Americans [0.002 (−0.002, 0.007)]. On a subgroup analysis stratified by BMI (underweight <18.5 kg/m^2^; normal, 18.5–24.9 kg/m^2^; overweight, 25–29.9 kg/m^2^; and obese, ≥30 kg/m^2^) (17), using the normal group as the reference group, the overweight group [0.010 (0.004, 0.016)] and obese group [0.006 (0.005, 0.008)] all maintained a significant positive relationship in Model 1, and the relationship between BMI and BMD was not statistically significant in the underweight group, although they had the highest beta values. In the overweight group, BMD increased by 0.010 g/cm^2^ per unit increase in BMI, while in the obese group, BMD increased by 0.006 g/cm^2^ per unit increase in BMI.

**Figure 2:**
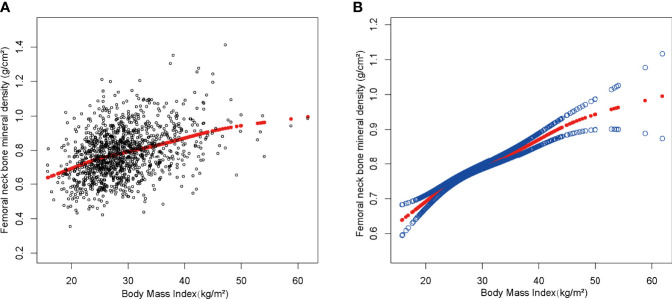
The association between body mass index and femoral neck bone mineral density. **(A)** Each black point represents a sample. **(B)** The solid red line represents the smooth curve fit between variables. Blue bands represent the 95% confidence interval from the fit. Age, gender, race, education level, activities status, diabetes status, NAFLD status, smoking status, ALT, ALP, AST, total calcium, total cholesterol, direct HDL cholesterol, LDL cholesterol, triglyceride, and serum phosphorus were adjusted.

We discovered a saturation effect value between BMI and BMD when we performed smoothing curve fitting in the amended model (**Figure 2**). We employed the saturation effect analysis model to look into the BMI turning point and discovered that the saturation effect value in the femoral neck BMD was 24.3 kg/m^2^ (**Table 4**). For each unit rise in BMI over 24.3 kg/m^2^, the femoral neck BMD rose by 0.015 g/cm^2^. When BMI exceeded 24.3 kg/m^2^, however, the femoral neck BMD increased only by 0.007 g/cm^2^ per unit rise in BMI.

### Relationship Between Waist Circumference and Femoral Neck BMD

**Table 3** represents the association between WC and femoral neck BMD for the three linear regression models. WC was significant positively linked with femoral neck BMD in the unadjusted model [0.004 (0.003, 0.004)], Model 2 [0.003 (0.003, 0.003)], and Model 3 [0.003 (0.003, 0.004)].

**Table 3:** Association between waist circumference (cm) and femoral neck bone Mineral density (g/cm^2^).

	Model 1*β* (95% CI) *p-*value	Model 2*β* (95% CI) *p-*value	Model 3*β* (95% CI) *p-*value
Waist circumference (cm)	0.004 (0.003, 0.004) <0.00001	0.003 (0.003, 0.003) <0.00001	0.003 (0.003, 0.004) <0.00001
Q1 (<91.3), *n* = 725	Reference	Reference	Reference
Q2 (91.4–99.9), *n* = 715	0.007 (0.003, 0.011) 0.00151	0.005 (0.001, 0.008) 0.01232	−0.000 (−0.006, 0.006) 0.99360
Q3 (100.0–110.1), *n* = 730	0.001 (−0.002, 0.005) 0.42547	0.001 (−0.002, 0.004) 0.42149	0.000 (−0.005, 0.005)0.92797
Q4 (>110.2), *n* = 733	0.003 (0.001, 0.004) 0.00003	0.002 (0.001, 0.003) 0.00045	0.003 (0.001, 0.004)0.00598
*p* for trend	<0.001	<0.001	<0.001
Subgroup analysis stratified by gender			
Male	0.003 (0.002, 0.003) <0.00001	0.003 (0.002, 0.003) <0.00001	0.003 (0.002, 0.004) <0.00001
Female	0.004 (0.003, 0.004) <0.00001	0.003 (0.003, 0.004) <0.00001	0.003 (0.002, 0.004) <0.00001
Subgroup analysis stratified by race			
Non-Hispanic White	0.003 (0.003, 0.004) <0.00001	0.003 (0.002, 0.003) <0.00001	0.003 (0.002, 0.004) <0.00001
Non- Hispanic Black	0.004 (0.003, 0.004) <0.00001	0.004 (0.003, 0.004) <0.00001	0.004 (0.003, 0.005) <0.00001
Mexican American	0.002 (0.001, 0.003) 0.00022	0.002 (0.001, 0.003) 0.00090	0.000 (−0.002, 0.002) 0.92756
Other race	0.004 (0.003, 0.005) <0.00001	0.003 (0.003, 0.003) <0.00001	0.003 (0.003, 0.004) <0.00001

Model 1: No covariates were adjusted.

Model 2: Age, gender, and race were adjusted.

Model 3: Age, gender, race, education level, activities status, diabetes status, NAFLD status, smoking status, ALT, ALP, AST, total calcium, total cholesterol, direct HDL cholesterol, LDL cholesterol, triglyceride, and serum phosphorus were adjusted.

In the subgroup analysis stratified by gender or race, the model is not adjusted for the stratification variable itself.

**Figure 3:**
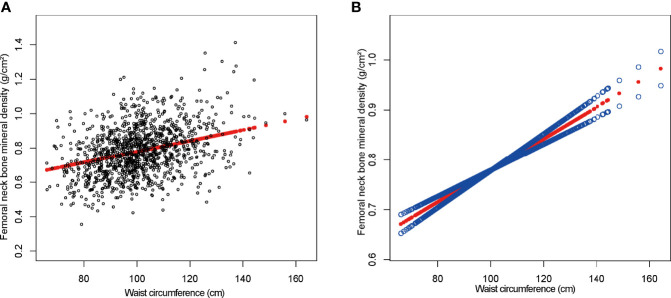
The association between waist circumference and femoral neck bone mineral density. **(A)** Each black point represents a sample. **(B)** The solid red line represents the smooth curve fit between variables. Blue bands represent the 95% confidence interval from the fit. Age, gender, race, education level, activities status, diabetes status, NAFLD status, smoking status, ALT, ALP, AST, total calcium, total cholesterol, direct HDL cholesterol, LDL cholesterol, triglyceride, and serum phosphorus were adjusted.

On a subgroup analysis according to sex, WC was positively linked to femoral neck BMD in both men and women in the fully adjusted model [0.003 (0.002, 0.004)] with same effect values and confidence intervals. On subgroup analysis according to race, the relationship was the same as in BMI and femoral neck BMD, and the positive association was retained in whites [0.003 (0.002, 0.004)], blacks [0.004 (0.003, 0.005)], and other race [0.003 (0.003, 0.004)] in the fully adjusted model. However, this association became insignificant (*p* = 0.92756) after adjusting all other covariates in Mexican Americans [0.000 (−0.002, 0.002)]. On a subgroup analysis stratified by WC quartiles, group Q1 served as the reference group and both group Q2 [0.007 (0.003, 0.011)] and group Q4 [0.003 (0.001, 0.004)] maintained a significant positive correlation in Model 1. The positive correlation between WC and BMD in group Q3 [0.001 (−0.002, 0.005)] was weak and insignificant. **Figure 3** depicted smooth curve fits and generalized additive models that were utilized to define the nonlinear connection between WC and femoral neck BMD. The saturation effect analysis of WC and BMD was not significant with LRT test = 0.245 (**Table 4**).

**Table 4:** Saturation effect analysis of BMI (kg/m^2^) and waist circumference (cm) on femoral neck BMD (g/cm^2^) of all participants.

Femoral neck bone mineral density	Model: Saturation effect analysis
BMI turning point (K), kg/m^2^	24.3
<K, effect 1	0.015 (0.009, 0.021) <0.0001
>K, effect 2	0.007 (0.006, 0.009) <0.0001
Effect 2 – 1	−0.008 (−0.014, −0.001) 0.0165
LRT test	0.015
Waist circumference (K), cm	95
<K, effect 1	0.004 (0.003, 0.005) <0.0001
>K, effect 2	0.003 (0.002, 0.004) <0.0001
Effect 2 – 1	−0.001 (−0.003, 0.001) 0.2499
LRT test	0.245

Age, gender, race, education level, activities status, diabetes status, NAFLD status, smoking status, ALT, ALP, AST, total calcium, total cholesterol, direct HDL cholesterol, LDL cholesterol, triglyceride, and serum phosphorus were adjusted.

## Discussion

In this study of individuals aged over 50 years, we demonstrated the positive association between BMI, WC and BMD. Of note, a BMI saturation value (24.3 kg/m^2^) was discovered in the femoral neck BMD in all subjects. At BMI levels <24.3 (kg/m^2^), the increase in BMD was greater as BMI increased, whereas at BMI levels above 24.3 (kg/m^2^), the increase was small, which is important for maintaining optimal BMD.

Osteoporosis and obesity have become serious health concerns across the world. The high frequency of these two disorders necessitates a deeper understanding of their link. Evidence on the link between WC and BMD has remained disputed to this point. In a recent cross-sectional study of 5,084 adults, Hua et al. found an inverse relationship between WC and BMD in middle-aged men with obesity (BMI ≥ 30 kg/m^2^) and overweight women (BMI < 25 kg/m^2^) (18). This conclusion is also supported by three cross-sectional studies from Asia (19–21). By comparison, in a cross-sectional study done in Turkey, WC was found to have a substantial positive relationship with total hip BMD, but a negative relationship with non-weight-bearing locations (22). These differences could be due to differences in study design, population, BMD quantification method, measurement site, or covariates. The WC has been used to assess abdominal obesity, whereas the BMI has been used to assess overall obesity. A cross-sectional research of the American population found a link between BMI and BMD (23). Another study by Wiacek et al. indicated that among Polish women aged 40–79 years, there was a significant positive correlation between BMI and BMD (24). Morin et al. noted that low BMI not only predicted the development of osteoporosis leading to osteoporosis, but also predicted an increased risk of fracture (25). A recent meta-analysis showed that adults with high BMI have higher lumbar BMD and femoral neck BMD compared to healthy weight individuals (26). BMI and WC are often congruent. However, people with normal BMI but large WC have a higher risk of developing metabolic diseases (27). A cohort study based on 44,366 women and men (mean age 70 years) in Sweden comprehensively assessed the relationship between body composition and fracture risk. The study found that fat distribution has a very strong effect on BMD and fracture risk and suggests that for optimal bone loss and fracture prevention, both men and women should avoid low BMI while having a high degree of central obesity (abdominal obesity).

In past studies, obesity has not only been shown to be associated with BMD, but has also been found to correlate with bone quality and fracture risk. Shen et al. studied 3,067 men in a cross-sectional research to determine the association between BMI and hip QCT measures, as well as to perform finite element analysis of hip QCT scans to offer a measure of hip strength during simulated falls. Men with obesity have better hip strength, but they also have a higher impact-to-strength ratio, which means that despite having stronger bones, they have a higher chance of hip fracture (28). The incidence of fracture declined with rising BMI and plateaued in men with obesity, according to a cohort study of 43,000 individuals aged 60 to 79 years from Norway. After adjusting for BMI and other possible confounders, larger WC and waist-to-hip ratio were linked to an increased risk of hip fracture. In fact, compared to men in the lowest tertile of WC, those in the highest tertile had a 100% increased risk of hip fracture. When a low BMI is combined with abdominal fat, the risk of hip fracture skyrockets (29). In 2005, a meta-analysis of 12 prospective population-based cohorts was released (approximately 60,000 individuals with a mean age of 62.2 years). Men and women with a low BMI had a greater age-corrected risk of any type of fracture, whereas those with a higher BMI had a lower risk. The increased risk, on the other hand, was not linear, and the gradient seemed steeper at lower BMI levels (30). This point is similar to the relationship between BMI, WC, and BMD in our study. The decreased bone mass and increased risk of fragility fractures associated with obesity also suggest that we need to keep BMI and WC in a reasonable range.

The mechanisms behind the relationship between obesity and BMD are unclear. Increased static mechanical compliance due to excessive fat accumulation is one of the hypothesized reasons. Increased static mechanical stresses on the skeleton are caused by excessive fat accumulation and body weight, and when bone tissue recognizes the mechanical forces imposed by the body, it undergoes a series of changes (31, 32). Another probable cause is the replacement of osteoblasts in the bone marrow by adipocytes. Because both osteoblasts and adipocytes are formed from mesenchymal stem cells in the bone marrow, enhanced lipogenic differentiation reduces osteogenic differentiation (33). In addition to the aforementioned two possibilities, another possibility is that obesity-induced hypermetabolism caused by increased insulin signaling causes bone marrow stromal stem cells to age more quickly (34). Furthermore, the more body fat a patient with obesity has, the higher the levels of different hormones such as estrogen (35) and insulin (36), which are helpful to BMD by blocking bone resorption and boosting bone remodeling (37, 38).

Nevertheless, when BMI exceeded a particular threshold of 24.3 (kg/m^2^), the femoral neck BMD increased by just 0.007 (g/cm^2^) per unit of BMI. The reasons for the saturating effect of BMI on BMD remain to be fully understood. Early in infancy, bone development trajectories and peak bone mass are defined, which might explain why adult BMD does not rise after a period of restricted growth (39, 40). Another cause for the occurrence of BMI saturation effects is a distinct bone–fat axis that exists *in vivo* between adipose and bone tissue (41), connected by various bioactive chemicals and maintaining bone homeostasis. According to existing research, bone and adipocytes are both derived from the same stem cell ancestor and are competitive, with an increase in excess fat leading to bone loss (42). BMD diminishes with growing obesity in animals with obesity, according to experiments in animal models caused by high-fat diets (43, 44).

Our findings are extremely relevant to the entire population since we used a nationally representative sample. We were also able to undertake subgroup analyses of BMI, WC, and femoral neck BMD across gender and ethnicity, and find the saturation effect value of obesity on BMD because of our large sample size. However, it is crucial to acknowledge the study’s limitations. The fundamental weakness of the study is its cross-sectional design. The causal relationship between BMI, WC, and femoral neck BMD could not be determined. To understand the specific mechanism of the relationship between obesity and BMD, further fundamental mechanistic research and large sample prospective studies are required. Second, due to database limitations, we were unable to identify participants with visceral fat, participants with fractures, or participants with osteoarthritis, as well as participants’ lumbar spine BMD data, so our findings should be viewed with caution. Third, our study lacked data on participants’ medications, such as calcium supplements and lipid-lowering drugs, which may also account for the differences in calcium and cholesterol levels in the baseline tables. Fourth, due to the limitations of the NHANES database for race-based classification criteria, we are unable to provide the percentage of abdominally obese individuals in each group for all race-specific thresholds. Finally, we excluded participants with cancer and our findings cannot be applied to a specific group.

## Conclusion

In conclusion, we discovered not only a significant positive connection between BMI, WC, and BMD, but also a BMI saturation value for femoral neck BMD. According to this study, maintaining BMI at a moderate level (about 24.3 kg/m^2^) would result in an optimal balance between BMI and BMD in adults over 50 years of age.

## Data Availability Statement

The original contributions presented in the study are included in the article/**Supplementary Material**, further inquiries can be directed to the corresponding author.

## Ethics Statement

The studies involving human participants were reviewed and approved by NCHS Ethics Review Board. The patients/participants provided their written informed consent to participate in this study.

## Author Contributions

YZ and JP designed the research. YZ collected and analyzed the data, and drafted the manuscript. YZ and JP revised the manuscript. All authors contributed to the article and approved the submitted version.

## Funding

This study was funded by the Guangxi Natural Science Foundation Project (2019GXNSFBA245023) and the Science and Technology Project, Guangxi, (AD17129025).

## Conflict of Interest

The authors declare that the research was conducted in the absence of any commercial or financial relationships that could be construed as a potential conflict of interest.

## Publisher’s Note

All claims expressed in this article are solely those of the authors and do not necessarily represent those of their affiliated organizations, or those of the publisher, the editors and the reviewers. Any product that may be evaluated in this article, or claim that may be made by its manufacturer, is not guaranteed or endorsed by the publisher.

## Abbreviations

BMD, bone mineral density; NAFLD, non-alcoholic fatty liver disease; BMI, body mass index; WC, waist circumference; NHANES, National Health and Nutrition Examination Survey; MEC, mobile examination center.
