# Supplementary material for: The Saturation Effect of Obesity on Bone Mineral Density for Older People: The NHANES 2017–2020
Source: Front Endocrinol (Lausanne). 2022 May 16;13:883862. doi: 10.3389/fendo.2022.883862 (PMC9150366; doi:10.3389/fendo.2022.883862)
Supplement: Supplementary file 1 [file DataSheet_1.docx]

| **Waist circumference (cm)** | **Normalweight** | **Overweight** | **Obese I** | **Obese II and III** | ***P* value** |
| --- | --- | --- | --- | --- | --- |
| **Females** | **（80-89）** | **（90-104）** | **（105-114）** | **（≥115）** |  |
| Total | 28.111 | 40.556 | 17.204 | 14.129 |  |
| *Race/Ethnicity (%)* |  |  |  |  | < 0.001 |
| Non-Hispanic White | 25.786 | 39.832 | 18.449 | 15.933 |  |
| Non-Hispanic Black | 18.644 | 38.418 | 20.056 | 22.882 |  |
| Mexican American | 20.313 | 50.781 | 18.750 | 10.156 |  |
| Other race/ethnicity | 41.523 | 40.049 | 12.776 | 5.652 |  |

**Supplementary Table S1:** Percentage of obesity based on waist circumference in women and men by race

| **Waist circumference (cm)** | **Normalweight** | **Overweight** | **Obese I** | **Obese II and III** | ***P* value** |
| --- | --- | --- | --- | --- | --- |
| **Males** | **（90-99）** | **（100-114）** | **（115-124）** | **（≥125）** |  |
| Total | 43.917 | 36.239 | 12.557 | 7.287 |  |
| *Race/Ethnicity (%)* |  |  |  |  | < 0.001 |
| Non-Hispanic White | 36.364 | 38.298 | 16.441 | 8.897 |  |
| Non-Hispanic Black | 45.330 | 34.396 | 12.074 | 8.200 |  |
| Mexican American | 38.509 | 41.615 | 14.907 | 4.969 |  |
| Other race/ethnicity | 53.810 | 33.571 | 7.390 | 5.229 |  |

(%) for categorical variables: the *P* value was calculated by the weighted chi-square test.

[1] Ross, R., Neeland, I.J., Yamashita, S. et al. Waist circumference as a vital sign in clinical practice: a Consensus Statement from the IAS and ICCR Working Group on Visceral Obesity. Nat Rev Endocrinol. 2020, 16, 177–189.
